# Using phase diagrams with microseeding to prepare crystal samples for advanced data collection techniques

**DOI:** 10.1063/4.0001164

**Published:** 2025-10-27

**Authors:** Patrick D Shaw Stewart, Stefan A `Kolek, Jack R Stubbs, Peter F M Baldock

**Affiliations:** 1 Douglas Instruments Ltd, Hungerford, Berkshire, United Kingdom; 2 University of Southampton, Southampton, Hampshire, United Kingdom

## Abstract

Serial data collection and microED techniques typically require “slurries” of tiny, well-ordered crystals [1]. Neutron diffraction requires very large single crystals. Making samples for these techniques is often a complex process requiring many rounds of optimization. To guide them in this task, protein crystallizers often keep a notional phase diagram in mind, which has four zones: an undersaturated zone where protein always remains in solution, a metastable zone where crystals will grow when seeds are added, a crystal nucleation zone where crystals appear spontaneously, and a protein precipitation zone. However, the shape of real-life phase diagrams can vary, making the interpretation of experimental results difficult. It is therefore very helpful to determine the phase diagrams of individual target proteins experimentally. Douglas Instruments, in collaboration with the University of Southampton, has introduced a simple and rapid method of generating custom phase diagrams using just 15 – 60 μL of protein [Fig. 1]. A simple approach uses the microbatch-under-oil method to avoid concentrating the sample drop (as would occur in a vapor diffusion setup), and by carrying out the same procedure with and without a seedstock, the metastable zone can be identified [2]. Moreover, advanced methods often require relatively large sample volumes, and microbatch can easily be scaled up to 50 μL or larger “batches” using robotics. A new variation of the method eliminates the need for oil by using a sitting drop setup, where solutions are dispensed to the reservoirs that exactly balance the concentrations of the drops. We present case studies where phase diagrams were used to increase control and crystal quality for routine and advanced data collection.

